# Effect of Transcutaneous Electrical Acupoint Stimulation on One-Lung Ventilation-Induced Lung Injury in Patients Undergoing Esophageal Cancer Operation

**DOI:** 10.1155/2020/9018701

**Published:** 2020-06-07

**Authors:** Fangchao Zhao, Zengying Wang, Chengyuan Ye, Jianming Liu

**Affiliations:** ^1^Department of Thoracic Surgery, Tangshan People's Hospital, North China University of Science and Technology, Tangshan 063000, China; ^2^Department of Clinical Medicine, North China University of Science and Technology, Tangshan 063000, China; ^3^Department of Cancer Comprehensive Therapy, Tangshan People's Hospital, North China University of Science and Technology, Tangshan 063000, China

## Abstract

**Objective:**

To investigate the effect of transcutaneous electrical acupoint stimulation (TEAS) on one-lung ventilation-induced injury in patients undergoing esophageal cancer operation.

**Methods:**

The participants (*n* = 121) were randomly assigned into TEAS and sham groups. The TEAS group was given transcutaneous electrical stimulation therapy. The acupoints selected were Feishu (BL13), Hegu (L14), and Zusanli (ST36) and were treated 30 minutes before induction of anesthesia; treatment lasts 30 minutes. The sham group was connected to the electrode on the same acupoints, but electronic stimulation was not applied. The levels of oxygenation index (PaO_2_/FiO_2_) and alveolar-arterial oxygen tension difference (A-aDO_2_) before one-lung ventilation (T1), 30 minutes after one-lung ventilation (T2), 2 hours after one-lung ventilation (T3), and 1 hour after the operation (T4) and the levels of serum tumor necrosis factor-*α* (TNF-*α*), interleukin-6 (IL-6), and interleukin-10 (IL-10) at T1, T2, T3, and 24 hours after the operation (T5) were taken as the primary endpoints. The incidence of postoperative pulmonary complications, removal time of thoracic drainage tube, and length of hospital stay were taken as the secondary endpoints.

**Results:**

Compared with that, in the sham group, the level of PaO_2_/FiO_2_ in the TEAS group was significantly increased at T2, T3, and T4, and the level of A-aDO_2_ was significantly reduced at T2 and T3 (*P* < 0.05). Besides, compared with that, in the sham group, the level of serum TNF-*α* at T2, T3, and T5, as well as the level of serum IL-6 at T3 and T5, was significantly reduced, whereas the level of serum IL-10 at T3 was significantly increased (*P* < 0.05). The incidences of pulmonary infection and pleural effusion in the TEAS group were significantly lower than that in the sham group, and the removal time of thoracic drainage tube and the length of hospital stay in the TEAS group were significantly shorter than that in the sham group (*P* < 0.05).

**Conclusions:**

TEAS could effectively increase the levels of PaO_2_/FiO_2_ and IL-10, reduce the levels of A-aDO_2_, TNF-*α*, and IL-6, and reduce the incidence of pulmonary complications. Moreover, it could also contribute to shorten the removal time of thoracic drainage tube and the length of hospital stay.

## 1. Introduction

Esophageal carcinoma is one of the malignant tumors with high morbidity and mortality in China, the preferred treatment of which is surgical treatment [1]. One-lung ventilation provides a good visual field and manipulation space for esophageal cancer operation [2]. However, as a kind of nonphysiological ventilation mode, one-lung ventilation could induce a lot of pathological and physical changes, eventually leading to the injuries of lungs and other organs [3, 4]. For example, pulmonary ischemia-reperfusion injury and ventilation/perfusion imbalance destroy the alveolar capillary barrier [5] and subsequently trigger the release of massive inflammatory factors. After the restoration of two-lung ventilation, the recruitment maneuvers of collapsed alveoli cause ischemia-reperfusion injury of lung tissue, aggravate the ischemia and anoxia of lungs, trigger the release of massive inflammatory factors [6, 7], and induce pulmonary inflammatory response, thereby leading to pulmonary injury and the increase of postoperative pulmonary complications [8].

During the thoracic surgery with one-lung ventilation, the blood flow volume of lungs in nonventilation side reduced to 20% to 25% of cardiac output due to the impacts of body position and pulmonary vasoconstriction in hypoxic condition [9]. Besides, this part of blood flow is in hypoxic status owing to poor oxygenation, which could trigger the release of inflammatory factors [10]. Lung tissues receive obvious mechanical stretch during the recruitment maneuvers of collapsed lung. The following changes including restoration of oxygenated function and opening of pulmonary vessels could induce pulmonary ischemia-reperfusion injury, accompanied with the production of massive oxygen free radicals [11], which lead to upregulated expression of multiple inflammatory factors and inflammatory mediators, systemic inflammatory response, cascade reaction of lung tissue, and aggravation of ischemia/hypoxia-induced lung injury [12].

Research suggests that electroacupuncture could alleviate the levels of inflammatory reaction by downregulating the serum levels of cytokines TNF-*α* and IL-6 [13]. It is found in some studies that electrical acupoint stimulation could activate the cholinergic anti-inflammatory pathway and reduce the release of multiple proinflammatory cytokines such as TNF-*α*, IL-1, and IL-6 through a series of intracellular signal transduction pathways, thus achieving the purpose of inhibiting local or systemic inflammatory response [14, 15]. Transcutaneous electrical acupoint stimulation (TEAS) is a type of acupuncture that combines the effect of transcutaneous electrical nerve stimulation (TENS) and acupoint therapy [16]. TEAS could protect the perioperative immune system through regulating the balance of T lymphocytes and the expression levels of related cytokines as well as transcription factors, thereby protecting lungs through inhibiting one-lung ventilation-induced inflammatory response [17]. However, relevant studies regarding these effects are relatively lacking.

We conducted this randomized controlled study primarily to examine the effects of TEAS on one-lung ventilation-induced lung injury in TEAS patients with esophageal carcinoma operation.

## 2. Clinical Data

Ethical approval was obtained from the Ethical Committee of Human Research of Tangshan People's Hospital, North China University of Science and Technology (file number: RMYY-YWLL-2017-1110). The study was performed in accordance with the Declaration of Helsinki and the guidelines on good clinical practice. Approval by the regional research ethics committee was obtained. All patients enrolled in the study signed informed consent.

One hundred eighty-seven patients diagnosed with esophageal carcinoma in Tangshan People's Hospital, North China University of Science and Technology, from March 2018 to August 2019, were recruited for this study. The patients were included if they met the following criteria: (1) ASA grade II to III; (2) ARISCAT scores greater than or equal to 45 points; (3) no histories of cardiovascular and cerebrovascular diseases as well as hematological diseases; and (4) good cognitive function and language ability. Conversely, the exclusion criteria were as follows: (1) patients diagnosed with moderate-to-severe pulmonary insufficiency before the operation; (2) patients with the histories of obstructive or restrictive pulmonary disease and pulmonary arterial hypertension; (3) patients with infection in the lung or other positions; (4) patients in whom the target acupoint stimulation site was infected, who had trauma, or in whom TEAS implementation was not appropriate to carry out for other reasons; (5) patients who had already underwent thoracotomy; and (6) patients who had uncontrolled hypertension, diabetes, history of epilepsy, were pregnant, lactating or had childbearing potential, or were enrolled in other clinical trials. Finally, 144 patients met the inclusion criteria, but 23 refused to participate in this study. Therefore, a total of 121 patients underwent radical surgery for esophageal carcinoma.

## 3. Treatment Methods

All the patients underwent routine 8-hour fasting and 2-hour drinking before the operation. Thirty minutes before the operation, 0.3 mg of scopolamine was given through intramuscular injection. Subsequently, the patients underwent other preoperative preparation, including open peripheral veins, intraoperative monitoring of the heart rate (HR), mean blood pressure (MAP), peripheral capillary oxygen saturation (SpO_2_) and bispectral index (BIS), radial artery puncture pressure measurement, and establishment of upper limb venous access, followed by intravenous injection of midazolam (0.1 mg/kg), propofol (1 to 2 mg/kg), fentanyl (3 *μ*g/kg), and vecuronium (0.1 mg/kg) for anesthesia induction. And then, double-lumen endotracheal tube was inserted. After confirming by fiberobronchoscopy, the endotracheal tube was located in good position and the anesthesia effect was satisfactory. After endotracheal intubation, two-lung ventilation was performed with the following parameters: tidal volume (VT) of 8 to 10 mL/kg, fraction of inspired oxygen (FiO_2_) of 100%, inhalation-to-expiration ratio of 1 : 1.5, respiratory frequency of 10 to 15/minute, and PETCO_2_ of 35 to 45 mmHg. Anesthesia was maintained by continuous inhalation of isoflurane as well as intermittent injection of fentanyl (1 to 2 *μ*g/kg) and vecuronium (0.05 mg/kg). The end-tidal carbon dioxide partial pressure (PETCO_2_) and peak airway pressure were monitored simultaneously. The respiratory parameters mentioned above remained unchanged during one-lung ventilation, and the orifice of the unventilated endotracheal tube was opened in the air, causing the operated-side pulmonary collapse. During the operation, the operated-side lung was re-expanded as required (pressure of 40 cm H_2_O, 10 to 15 seconds each time). The airway was cleaned, and two-lung ventilation was restored when the chest was closed. The double-lumen endotracheal tube was removed after the patient was awake, and the ventilation index was satisfactory.

Patients in group TEAS received acupoint electrical stimulation on bilateral Feishu (BL13, located under the spinous process of the third thoracic vertebrae approximately 5 cm apart), Hegu (L14, between the first and second metacarpal bones on the dorsum of the hand, the midpoint of the radial side of the second metacarpal bone), and Zusanli (ST36, located 5 mm below and lateral to the anterior tubercle of the tibia) 30 minutes before induction of anesthesia. The HANS LH-202 electrical stimulator (Nanjing Ji Sheng Medical Technology Co, Ltd, Nanjing, China) was used to provide electrical stimulation. After skin disinfection, electrode tabs were placed on bilateral BL13, L14, and ST36.

The instrument parameters were set as follows: pulse width of 100 *μ*s, frequency of 100 Hz, stimulation duration of 10 seconds, stimulation interval of 3 seconds, stimulation amount of 20 to 25 mA, and maximum feedback stimulation amount of 40 mA. The intensity was set to the highest level that the patient could withstand.

For patients in group sham TEAS, electrode tabs were placed on bilateral BL13, L14, and ST36 similar to patients in group TEAS, but electrical stimulation was not initiated. The electrodes were well protected from detaching during the operation. The TEAS intervention was performed by a research nurse who was a qualified member of the research team and then verified by 2 traditional Chinese medicine physicians. Throughout the trial, participants were treated separately to prevent communication. We followed the methods given by [18].

## 4. Efficacy Observation

The primary endpoints were the levels of PaO_2_/FiO_2_, A-aDO_2_, TNF-*α*, IL-6, and IL-10. All the patients underwent blood gas analysis through radial blood specimen collection before one-lung ventilation (T1), 30 minutes after one-lung ventilation (T2), 2 hours after one-lung ventilation (T3), and 1 hour after the operation (T4) to calculate the levels of PaO_2_/FiO_2_ and A-aDO_2_. Internal jugular vein specimen collection was performed at T1, T2, T3, and 24 hours after the operation (T5) to measure the levels of TNF-*α*, IL-6, and IL-10 through enzyme linked immunosorbent assay (ELISA) (Shanghai Westang Biotechnology Co. Ltd., Shanghai, China). The measurement process was completed in clinical laboratory, following the manufacturer's description. The laboratory physicians were blinded to the work of research team. The secondary endpoints were the incidence of postoperative pulmonary complications (pulmonary infection, pleural effusion, and pulmonary atelectasis), removal time of thoracic drainage tube, and the length of hospital stay.

## 5. Statistical Analysis

Data were processed using SPSS, version 22.0 (SPSS Inc, Chicago, Illinois). Data are presented as the means and standard deviations for continuous variables and as proportions for categorical variables. Normally distributed continuous data (determined by the Kolmogorov–Smirnov method) were compared using Student's *t*-test. The *χ*^2^ test was used to analyze categorical variables. One-way analysis of variance was used to analyze differences between the baseline values and other time points. Repeated measurements were used to analyze differences in the interaction effects between groups and different time points. A 2-tailed *P* value of <0.05 was considered statistically significant.

## 6. Results

### 6.1. Participant Enrollment

**Figure 1:**
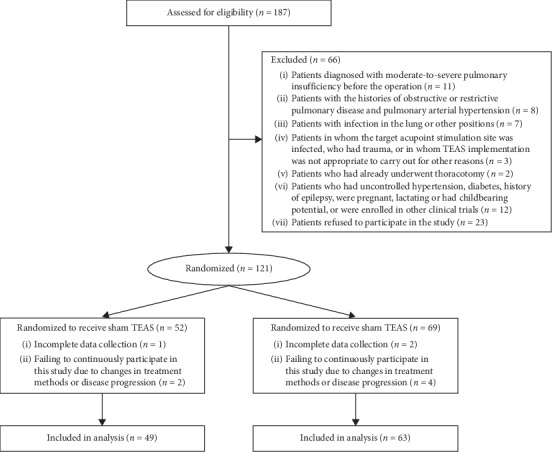
Flow of participants randomized to receive TEAS or sham TEAS. TEAS, transcutaneous electrical acupoint stimulation.

Of the 121 patients, 9 were excluded for the following reasons: incomplete data collection (*n* = 3) and failing to continuously participate in this study due to changes in treatment method or disease progression (*n* = 6). Finally, a total of 112 patients completed this study, including 49 patients from the sham group and 63 from the TEAS group (Figure 1). There was no significant difference in demographic characteristics and surgical information between both groups (*P* > 0.05; Table 1).

### 6.2. Levels of PaO_2_/FiO_2_ and A-aDO_2_

**Figure 2:**
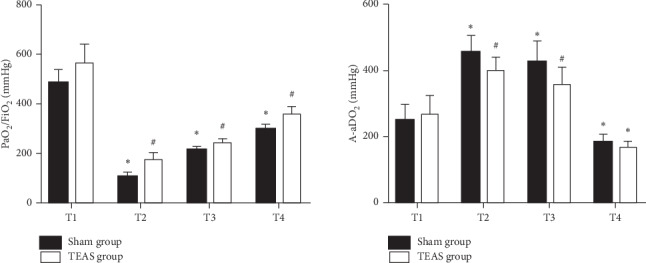
Levels of PaO_2_/FiO_2_ and A-aDO_2_ between 2 groups. ^*∗*^*P* < 0.05 versus group sham transcutaneous electrical acupoint stimulation (TEAS), ^#^*P* < 0.05 versus T1. T1, before one-lung ventilation; T2, 30 minutes after one-lung ventilation; T3, 2 hours after one-lung ventilation; T4, 1 hour after the operation.

The baseline levels of PaO_2_/FiO_2_ and A-aDO_2_ at T1 were comparable between both groups (*P* > 0.05). Compared with that at T1, the level of PaO_2_/FiO_2_ in both groups decreased at T2, T3, and T4, while the level of A-aDO_2_ increased at T2 and T3 but decreased at T4 (*P* < 0.05); compared with that in the sham group, the level of PaO_2_/FiO_2_ in the TEAS group increased at T2, T3, and T4, while the level of A-aDO_2_ decreased at T2 and T3 (Figures 2(a) and 2(b)).

### 6.3. Serum Levels of TNF-*α*, IL-6, and IL-10

**Figure 3:**
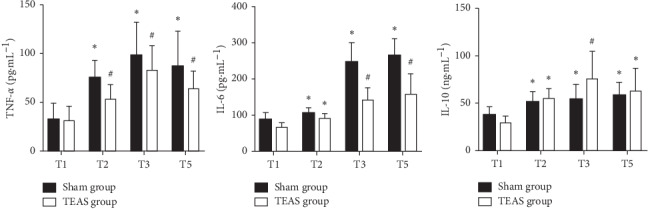
Serum levels of TNF-*α*, IL-6, and IL-10 between 2 groups. ^*∗*^*P* < 0.05 versus group sham transcutaneous electrical acupoint stimulation (TEAS), ^#^*P* < 0.05 versus T1. T1, before one-lung ventilation; T2, thirty minutes after one-lung ventilation; T3, two hours after one-lung ventilation; T5, twenty-four hours after the operation.

The baseline serum levels of TNF-*α*, IL-6, and IL-10 at T1 were comparable between both groups (*P* > 0.05). Compared with that at T1, the serum levels of TNF-*α*, IL-6, and IL-10 in both groups at T2, T3, and T5 increased to different degrees (*P* < 0.05). Compared with that in the sham group, the serum level of TNF-*α* in the TEAS group decreased at T2, T3, and T5, the level of IL-6 decreased at T3 and T5, and the level of IL-10 increased at T3 (*P* < 0.05; Figures 3(a)–3(c)).

### 6.4. Postoperative Complications

There was no significant difference on the incidence of pulmonary atelectasis between both groups. Compared with that in the sham group, the incidence of pulmonary infection and pleural effusion was significantly decreased; moreover, the removal time of thoracic drainage tube and the length of hospital stay were also significantly shortened (Table 2).

## 7. Discussion

One-lung ventilation, a common ventilation method for airway management during thoracic anesthesia, could make operated-side lung tissue completely collapse rather than ventilate through surgery to accomplish the procedure of anesthesia. Not only could it block the access of operated-side secretions and blood to the healthy-side lung so as to prevent the cross-infection and metastasis between operated-side lung and healthy-side lung, but also it could provide a clear and quiet visual field for esophageal carcinoma operation. Thus, it has become a main ventilation mode applied in esophageal carcinoma operation [19, 20]. However, everything has its pros and cons, and one-lung ventilation is no exception. During the period of one-lung ventilation, a series of factors, including the irritative injury of trachea and main bronchus caused by the thick double-lumen bronchial catheter, alveolar overexpansion in the ventilated lung, and injuries of the nonventilated lung through a complex process of alveolar collapse/pulmonary atelectasis, hypoxic pulmonary vasoconstriction, and reperfusion after the restoration of ventilation, could cause pulmonary mechanical and biochemical injuries such as oxidative stress, thereby leading to the release of large amounts of inflammatory cytokines [12]. The latter could induce pulmonary inflammation and cause lung injury, followed by increased postoperative pulmonary complications [21, 22]. As a consequence, it is of great importance to prevent and alleviate one-lung ventilation-induced inflammation response, minimize lung injury, reduce postoperative pulmonary complications, and improve the prognosis.

As a new kind of acupuncture therapy combining with transcutaneous electrical nerve stimulation and acupoint treatment, TEAS has the effects similar to those of electroacupuncture. An animal experiment showed that electroacupuncture stimulation at BL13 and Zusanli (ST36) acupoints could protect the lung through effectively regulating inflammatory responses [23]. Luo et al. proposed that electroacupuncture stimulation at BL13 acupoint could downregulate the lung index and serum TNF-*α* level in viral pneumonia mice and upregulate the serum IL-10 level [24]. Another study [25] revealed that stimulation at ST36 acupoint was able to significantly reduce the levels of pulmonary inflammatory mediators including TNF-*α*, IL-6, and IL-8, obviously increase the level of IL-10, diminish the edema, hemorrhage, and neutrophil infiltration in the pulmonary interstitium and alveoli, and inhibit the reduction of the pulmonary oxygenation index, which had a protective effect on lung injury caused by one-lung ventilation. Accordingly, BL13, LI4, and ST36 were selected as target acupoints for electrical stimulation in our study.

In this study, the oxygenation index (PaO_2_/FiO_2_) was decreased in both groups after the start of one-lung ventilation. Compared with that of the sham group, PaO_2_/FiO_2_ in the TEAS group increased at T2 to T4, and A-aDO_2_ reduced at T2 and T3 (*P* < 0.05). The above results indicated that TEAS could reduce ventilation/perfusion imbalance, decrease pulmonary shunt, and improve the oxygenation. Some studies showed that during perioperative period, TEAS was able to reduce ventilation/perfusion imbalance, repair hypoxic pulmonary vasoconstriction, and slow down the rate of PaO_2_ decline in the course of one-lung ventilation, which was in support of this study. The possible mechanism is that Kongzui and Feishu acupoints could effectively regulate pulmonary blood flow and lung ventilation, respectively. TEAS on Kongzui and Feishu acupoints will improve the ventilation/perfusion ratio, thereby reducing the rate of pulmonary shunt and promoting pulmonary oxygenation [26].

In the mechanism of one-lung ventilation-induced lung injury, the inflammatory mediators such as TNF-*α*, IL-6, and IL-10 exert an important regulatory role in the development and progression of inflammation [8]. IL-6 is not only the crucial factor for many inflammatory responses but also the marker of lung injury caused by operation and one-lung ventilation. Besides, it is also an early sensitive indicator for tissue damage [27]. Acting as a protective factor in inflammatory response, IL-10 is able to inhibit the synthesis of proinflammatory cytokines such as TNF-*α* and IL-6, prevent from the development and progression of inflammatory response, and alleviate the inflammatory injury [28]. TNF-*α*, one of the earliest released and most sensitive inflammatory cytokines, is an important proinflammatory factor in the development and progression of acute lung injury, which could directly damage pulmonary vascular endothelial cells and pulmonary surfactants, thereby resulting in acute lung injury [29]. It thus appears that the changes in the levels of TNF-*α*, IL-6, and IL-10 can contribute to evaluate the inflammation balance state and pulmonary injury. In this study, compared with that at T1, the levels of TNF-*α*, IL-6, and IL-10 in both groups increased in different degrees at T2, T3, and T5 (*P* < 0.05). Compared with that in the sham group, the levels of TNF-*α* and IL-6 in the TEAS group at T2, T3, and T5 decreased, while the level of IL-10 increased at T3 (*P* < 0.05). The above results suggest that TEAS could alleviate one-lung ventilation-induced lung injury and postoperative early inflammatory response. It is demonstrated that the levels of inflammatory cytokines during one-lung ventilation and postoperative early inflammatory response were closely associated with the development of postoperative pulmonary complications. Additionally, pulmonary inflammatory cells and inflammatory cytokines could also enter the systemic circulation via the damaged alveolar capillary barrier, thereby mediating the systemic inflammatory responses. Another study indicated that TEAS was able to alleviate the inflammatory response induced by lipopolysaccharide. In this study, there was no significant difference on the incidence of pulmonary atelectasis in both groups, while the incidences of pulmonary infection and pleural effusion in the TEAS group were significantly lower than that in the sham group. Besides, the removal time of thoracic drainage tube and the length of hospital stay in the TEAS group were shorter than that in the sham group, indicating that TEAS could contribute to reduce postoperative pulmonary complications, accelerate postoperative recovery, and improve prognosis.

## 8. Limitations

All patients were told that TEAS was carried out throughout the surgery. In addition, patients were treated separately to prevent communication throughout the trial and to reduce the bias of blinding as much as possible. The study was supposed to use absolute time, which would improve the objectivity of data collection and the reliability of results. We did not follow the long-term prognosis or tumor-free survival periods of the participants in our study. The long-term assessment of tumor progression after surgery is an important focus, and we recorded only the length of hospital stay in the current study. In future studies, larger sample-sized, double-blind multicenter randomized controlled trials with a long-term assessment of tumor progression after surgery are warranted to gather more evidence on the detailed effects of TEAS on the immunological function of patients with cancer.

## 9. Conclusion

The application of TEAS could effectively increase the levels of PaO_2_/FiO_2_ and IL-10, reduce the levels of A-aDO_2_, TNF-*α*, and IL-6, and reduce the postoperative pulmonary complications. Moreover, it is also beneficial to shorten the removal time of thoracic drainage tube so as to shorten the length of hospital stay.

## Figures and Tables

**Table 1:** Patient and surgical characteristics^a^.

Index	Group sham (*n* = 49)	Group TEAS (*n* = 63)	*χ* ^2^	*P* value
Gender			1.963	0.375
Male	39	48		
Female	10	15		
Age (years)	62.3 ± 7.2	63.1 ± 8.1	0.282	0.755
Smoking history	27	36	1.819	0.174
BMI (kg/m^2^)	24.6 ± 4.0	22.3 ± 5.7	1.867	0.276
ASA rating			0.634	0.702
II	37	49		
III	12	14		
ARISCAT score	51.7 ± 4.2	53.2 ± 5.5	1.053	0.418
Lung function			0.536	0.481
FEV1%	86.7 ± 12.4	83.1 ± 11.8		
FEV1/FVC	84.3 ± 9.0	83.5 ± 8.9		
Operation time (min)	213.2 ± 28.3	225.5 ± 31.5	0.217	0.641
Anesthesia time (min)	250.8 ± 47.6	271.7 ± 66.2	0.053	0.818
Single-lung ventilation time (min)	171.9 ± 17.6	188.1 ± 20.4	0.213	0.854
Intraoperative fluid volume (mL)	2280 ± 101	2120 ± 130	6.616	0.037
Amount of bleeding	137.4 ± 61.8	130.1 ± 46.6	0.556	0.634
TNM stage			1.495	0.221
I	7	8		
II	33	42		
III	9	13		

ASA, American Society of Anesthesiologists; BMI, body mass index (calculated as weight in kilograms divided by height in meters squared); TNM, tumor, node, and metastasis. ^*a*^Values are shown as mean (SD) or number of patients.

**Table 2:** Postoperative complications^a^.

Complication	Group sham (*n* = 49)	Group TEAS (*n* = 63)	*χ* ^2^	*P* value
Lung infection	15 (30.6)	6 (9.5)	5.257	0.042^#^
Pleural effusion	37 (75.5)	7 (11.1)	8.613	0.002^#^
Atelectasis	4 (8.1)	2 (3.2)	0.182	0.670
Thoracic drainage tube removal time	8.1 ± 0.9	7.2 ± 0.6	7.323	0.028^#^
Length of hospital stay	15.5 ± 1.3	13.8 ± 0.4	7.625	0.014^#^

TEAS, transcutaneous electrical acupoint stimulation. ^#^*P* < 0.05 versus group sham TEAS. ^*a*^Values are number (proportion).

## Data Availability

The data sets used and/or analyzed during the current study are available from the corresponding author upon reasonable request.

## Abbreviations

HR: Heart rate
MAP: Mean arterial pressure
VT: Volume tidal
BIS: Bispectral index
TEAS: Transcutaneous electrical acupoint stimulation
TNF: Tumor necrosis factor
IL: Interleukin
ASA: American Society of Anesthesiologists
BMI: Body mass index
TNM: Tumor, node, and metastasis.
